# Effect of Stocking Rate on Soil-Atmosphere CH_4_ Flux during Spring Freeze-Thaw Cycles in a Northern Desert Steppe, China

**DOI:** 10.1371/journal.pone.0036794

**Published:** 2012-05-08

**Authors:** Cheng-Jie Wang, Shi-Ming Tang, Andreas Wilkes, Yuan-Yuan Jiang, Guo-Dong Han, Ding Huang

**Affiliations:** 1 College of Ecology and Environmental Science, Inner Mongolia Agricultural University, Huhhot, China; 2 World Agroforestry Centre, 12 Zhongguancun, Beijing, China; 3 Institute of Grassland Science, China Agricultural University, Beijing, China; Ohio State University, United States of America

## Abstract

**Background:**

Methane (CH_4_) uptake by steppe soils is affected by a range of specific factors and is a complex process. Increased stocking rate promotes steppe degradation, with unclear consequences for gas exchanges. To assess the effects of grazing management on CH_4_ uptake in desert steppes, we investigated soil-atmosphere CH_4_ exchange during the winter-spring transition period.

**Methodology/Main Finding:**

The experiment was conducted at twelve grazing plots denoting four treatments defined along a grazing gradient with three replications: non-grazing (0 sheep/ha, NG), light grazing (0.75 sheep/ha, LG), moderate grazing (1.50 sheep/ha, MG) and heavy grazing (2.25 sheep/ha, HG). Using an automatic cavity ring-down spectrophotometer, we measured CH_4_ fluxes from March 1 to April 29 in 2010 and March 2 to April 27 in 2011. According to the status of soil freeze-thaw cycles (positive and negative soil temperatures occurred in alternation), the experiment was divided into periods I and II. Results indicate that mean CH_4_ uptake in period I (7.51 µg CH_4_–C m^−2^ h^−1^) was significantly lower than uptake in period II (83.07 µg CH_4_–C m^−2^ h^−1^). Averaged over 2 years, CH_4_ fluxes during the freeze-thaw period were −84.76 µg CH_4_–C m^−2^ h^−1^ (NG), −88.76 µg CH_4_–C m^−2^ h^−1^ (LG), −64.77 µg CH_4_–C m^−2^ h^−1^ (MG) and −28.80 µg CH_4_–C m^−2^ h^−1^ (HG).

**Conclusions/Significance:**

CH_4_ uptake activity is affected by freeze-thaw cycles and stocking rates. CH_4_ uptake is correlated with the moisture content and temperature of soil. MG and HG decreases CH_4_ uptake while LG exerts a considerable positive impact on CH_4_ uptake during spring freeze-thaw cycles in the northern desert steppe in China.

## Introduction

Methane (CH_4_) is the second most important long-living greenhouse gas (GHG) after carbon dioxide (CO_2_). It is widely recognized that an increases in atmospheric CH_4_ concentration may cause global warming. The Intergovernmental Panel on Climate Change (IPCC) reported that global warming potential-weighted emissions of GHG increased by approximately 70% from 1970 to 2004, including emissions of CH_4_ which have risen by about 40%. The global warming potential of CH_4_ over a 100-year timeframe is estimated at 25 times that of CO_2_ [1].

Chinese grasslands cover 41.7% of China's land area, and are distributed mainly in Inner Mongolia, Xinjiang, Gansu, and the Qinghai-Tibet plateau [2]. Approximately 1.42 million ha of grassland in Inner Mongolia is classified as desert steppe, accounting for 18% of the total steppe area in China [3]. In recent decades, long-term high intensity grazing has led to severe degradation and desertification in this natural grassland ecosystem, with notable impacts on agricultural activities. Steppe soils are known to function as a significant sink for atmospheric CH_4_ [4]. It has been reported that in Inner Mongolian grasslands grazing changes soil moisture holding capacity, which in turn affects GHG emissions [4], [5], [6]. However, most previous studies reported measurements carried out on typical grassland during the growing season. Few studies have addressed CH_4_ uptake during the freeze-thaw period [7]. Mosier et al. (1996) reported for North American prairie systems that winter fluxes may contribute 30–40% of the annual N_2_O and CH_4_ fluxes [8]. Therefore, current estimates for annual exchange rates of CH_4_ between steppe soils and the atmosphere are still uncertain. To combat this uncertainty, CH_4_ exchange should be observed during the winter and winter-spring transition period in Inner Mongolian steppes.

Soil freezing and thawing events affect the soil physical structure and solute distribution as well as nutrient availability. This causes secondary effects on microorganism activity [7]. Nutrient and moisture conditions in frozen soil not only allow physiological activity of microorganisms even under strong frost conditions [9], and can also greatly promote their activity, especially during the spring thaw period [7]. The desert steppe is vulnerable to the impacts of livestock because of its relatively short and sparse ground cover and sandy soil. Wolf et al. (2010) reported that increased stocking rates in a continental steppe led to reductions in natural N_2_O release during the spring thawing period [6], but the impacts of grazing on CH_4_ exchange remain unknown, especially in the Inner Mongolian desert steppe. Our aim here was to investigate the characteristics of soil-atmosphere CH_4_ exchange across a gradient of stocking rates during the winter-spring transition, and to thus answer whether stocking rates influence CH_4_ fluxes in the desert steppe ecosystem in Inner Mongolia. We hypothesize: 1) grazing management affects CH_4_ uptake capacity of steppe soils during the spring thawing period, and 2) the moisture content and temperature of soil are the principal factors controlling CH_4_ uptake.

## Materials and Methods

### Study site description

The study was conducted at an experimental site at the Inner Mongolia Academy of Agriculture and Animal Husbandry Research Station (41°47′17″N, 111°53′46″E). The site has an elevation of 1450 m and is in a temperate continental climate, characterized by a short growing season and long cold winter with a frost-free period of 175 days. January is the coldest month with an average temperature of −15.1°C while July is the warmest month with an average temperature of 19.6°C. Average annual precipitation is approximately 280 mm, of which nearly 75% falls during June through September. The grassland is dominated by *Stipa breviflora* Griseb., *Artemisia frigida* Willd., *Cleistogenes songorica* (Roshev.) Ohwi, and accompanied by *Convolvulus ammannii* Desr., *Heteropappus altaicus* (Willd.) Novopokr., *Neopallasia petinata* (Pall.) Poljak., *Bassia prostrate* (L.) A.J. Scott, *Caragana stenophylla* Pojark., *Leymus chinensis* (Trin.) Tzvelev. The dominant soil types are Kastanozem (FAO soil classification) or Brown Chernozem (Canadian Soil Classification) with a loamy sand texture [10].

### Measurements of CH_4_ and other factors

CH_4_ fluxes were measured in twelve grazing plots during March 1–April 29 in 2010 and March 2–April 27 in 2011. The areas are also used for a grazing experiment started in 2002 with four stocking rates (non-grazing, 0 sheep/ha, NG; light grazing, 0.75 sheep/ha, LG; moderate grazing, 1.50 sheep/ha, MG; and heavy grazing, 2.25 sheep/ha, HG) with three replications each. The areas have been grazed from June to October only. The stocking rate was calculated on the basis of species composition and ground cover. The main characteristics of the grazing areas are shown in Table 1. CH_4_ fluxes in each plot with three fixed points were measured at the same time (i.e. 10:30 to 12:30) at an interval of one day, using an automatic cavity ring-down spectrophotometer (Picarro G1301, Santa Clara, CA, USA). The principle of the measurement is wavelength scanning optical cavity ring-down spectroscopy (WS-CRDS) technology. CH_4_ fluxes were calculated according to the following equation:
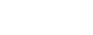
where *F* is the flux (mg m^−2^ h^−1^) of gas; *ρ* is the density of gas; *ΔC/ΔT* is the slope of the linear regression for gas concentration gradient through time, negative values indicating CH_4_ uptake into soil from atmosphere; *V* and *A* are volume (m^3^) and the hood base area (m^2^), respectively.

**Table 1:** Main characteristics of studied grazing areas.

Items	NG	LG	MG	HG
Plots sizes (ha)	4.0	4.0	4.0	4.0
Stocking rate (sheep ha^−1^)	0.00	0.75	1.50	2.25
Vegetation characteristics (growing season)				
Aboveground biomass (g m^−2^, DM)	57.84	45.13	27.24	13.14
Plant cover (%)	21	22	19	18
Soil characteristics				
Bulk density, 0–10 cm (g cm^−3^)	1.23	1.29	1.31	1.38
C to N ratio, 0–10 cm	7.75	7.82	7.75	6.83
Organic C content, 0–10 cm (g kg^−1^)	13.09	12.91	12.55	11.68
Soil texture, 0–10 cm				
Sand (%)	59.2	63.8	69.3	69.1
Silt (%)	29.7	26.9	20.1	19.8
Clay (%)	11.0	9.3	10.6	11.1

NG, non-grazing; LG, light grazing; MG, moderate grazing; HG, heavy grazing.

Soil temperature (5 cm depth) and moisture (0–5 cm) were measured by thermocouples and a hand-held reader (HH-25TC, OMEGA Engineering Inc., Stamford, CT) and a portable TDR probe (HH2, Delta-T Devices, Cambridge, UK) at the same time as the measurement of CH_4_ flux.

### Partition of the measurement period

**Figure 1:**
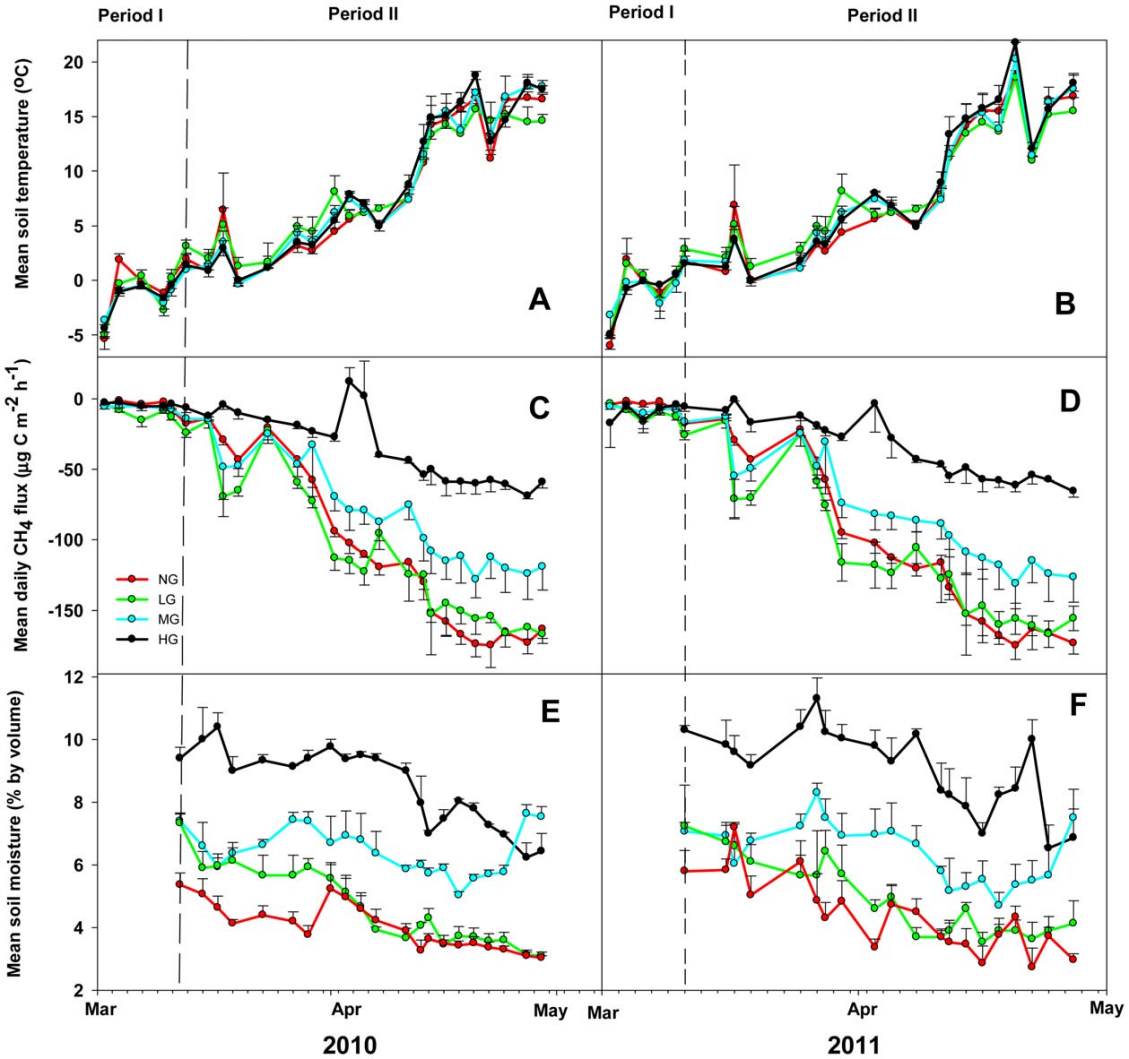
CH_4_ fluxes, soil temperature and soil moisture (5 cm depth) under different stocking rates. Measurement periods were from March 1 to April 27 in 2010 and March 2 to April 27 in 2011. The left side of the dashed line in the graph indicates Period Ι (March 1–12 in 2010 and March 2–10, 2011), and the right part period II (March 15 to April 29 in 2010 and March 11 to April 27 in 2011) (NG, non-grazing; LG, light grazing; MG, moderate grazing; HG, heavy grazing). The treatment symbols represent mean values for three plots (9 points). A pertain to soil temperature in 2010, B soil temperature in 2011, C CH_4_ fluxes in 2010, D CH_4_ fluxes in 2011, E soil moisture in 2010, and F soil moisture in 2011.

Soil temperature remained below zero and was characterized by severe frost from March 1 to March 12 in 2010 and from March 2 to March 10 in 2011. Therefore, soil moisture could not be measured due to the frozen topsoil. This period is referred to as period I. Plants started germination soon after positive and negative soil temperatures occurred in alternation, and the soil became unfrozen down to a depth of 5 cm (Figure 1A, B), as freeze-thaw events occurred. This period is referred to as period II.

### Statistical analysis

CH_4_ fluxes were analyzed using MIXED procedure of the Statistical Package for Social Science (SPSS 13.0 for Windows, 2003) [11], to test experimental different in CH_4_ fluxes. Replicate flux measurements were averaged by the sampling point for each grazing plot. Stocking rate, year, period and all possible interactions were treated as fixed effects, with the grazing plot of each stocking rate as a random effect, and sampling date as the repeated measure with the grazing plot used as the subject. The best fit covariance structure was determined to be compound symmetry. The data was examined for homogeneity of variances (Levene statistic test) and for normal distribution (Kolmogorov-Smirnov test) before analysis. All data was adjusted using log transformation (+1) after test. This transformation is usually applied when observed values were zero. The model testing interactive effects of stocking rate, period and year for CH_4_ flux and their degrees of freedom are shown in Table 2. Paired means of significant differences in treatments were determined using Fisher's least significant difference (LSD) statistic. To test the correlations between soil temperature, moisture and CH_4_ fluxes, Pearson's correlation analysis was performed. Linear and quadratic regression analysis was used to test the possible dependency of CH_4_ fluxes on soil moisture and temperature. R^2^ (square of Pearson correlation coefficient) value was used to decide the best fitted function (linear or quadratic). All significances mentioned in this paper are at the P = 0.05 level.

**Table 2:** MIXED model testing interactive effects of stocking rate, period and year for CH_4_ flux and their degrees of freedom.

Effect	Degrees of freedom	P value
Stocking rate (S)	3	<0.0001
Period (P)	1	<0.0001
Year (Y)	1	0.646
S×P	3	<0.0001
S×Y	3	0.941
P×Y	1	0.608
S×P×Y	3	0.998

## Results

### Effect of freeze-thaw events on CH_4_ exchange

**Figure 2:**
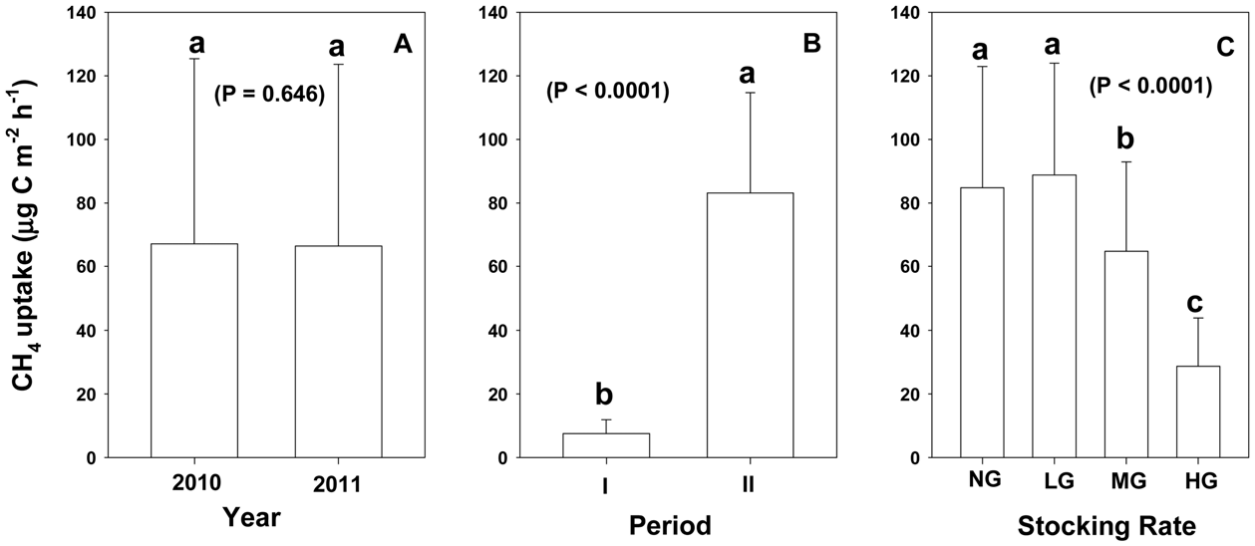
Comparison of CH_4_ uptake in desert steppes during the spring thaw period. The data shown in (A) represent the mean values for two years at different sites. The data illustrated in (B) are mean values for two periods at different sites. The data shown in (C) represent the mean values for four treatment groups. Different lowercase letters indicate significant differences among groups. NG, LG, MG, and HG are non-grazing, light grazing, moderate grazing, and heavy grazing, respectively.

Soil CH_4_ fluxes were not affected (P = 0.646) by year in our study. Averaged over 2 years, CH_4_ fluxes in the freeze-thaw period were −67.13 µg CH_4_-C m^−2^ h^−1^ and −66.40 µg CH_4_-C m^−2^ h^−1^ for periods I and II respectively (Figure 2A).

CH_4_ uptake of soil was affected by freeze-thaw events. Mean CH_4_ fluxes were significantly increased in period ΙI (−83.07 µg CH_4_-C m^−2^ h^−1^) compared to period I (−7.51 µg CH_4_-C m^−2^ h^−1^) (Figure 2B). The soil surface was covered with patched snow until the start of period II. The soil surface of the NG and LG plots even had a thin layer of ice derived from thaw-refreezing that could be observed during period I. We did not measure CH_4_ fluxes in the snow and ice sites for this experiment. Therefore, it remains unclear whether these snow patches and ice layers affect CH_4_ fluxes.

### Influence of stocking rates on CH_4_ exchange

The measured mean CH_4_ fluxes for NG, LG, MG and HG plots in 2010 and 2011 were shown in Table 3. CH_4_ fluxes during the freeze-thaw period, averaged over 2 years, were illustrated in Figure 2C. The strength of soil-atmospheric CH_4_ uptake decreased with an increase in stocking rate and the grazing areas were a CH_4_ sink during the entire period investigated. The uptake and emission of CH_4_ ranged from −197.93 to 42.03 µg CH_4_-C m^−2^ h^−1^ in the measured period. The main uptake of CH_4_ occurred at the NG and LG plots indicating a strong sink (Figure 1C, D). CH_4_ uptake in LG plots was higher compared to uptake in the other plots during period Ι, and uptake of NG and LG plots was higher compared to MG and HG plots during period II (Table 3).

**Table 3:** Mean fluxes of CH_4_ (µg CH_4_-C m^−2^ h^−1^) under different stocking rates during spring freeze-thaw period.

Period	2010				2011			
	NG	LG	MG	HG	NG	LG	MG	HG
I	−6.04	−11.95	−7.37	−4.24	−3.95	−9.86	−7.20	−9.46
II	−110.24	−112.55	−82.02	−35.38	−103.10	−107.74	−79.13	−34.43
I+II	−86.19	−89.33	−64.79	−28.19	−83.27	−88.16	−64.74	−29.44

Period Ι: March 1–12 2010 and March 2–10 2011; period II: March 15 to April 29 2010 and March 11 to April 27 2011.

NG, non-grazing; LG, light grazing; MG, moderate grazing; HG, heavy grazing.

### Relationship between CH_4_ uptake and other factors

**Figure 3:**
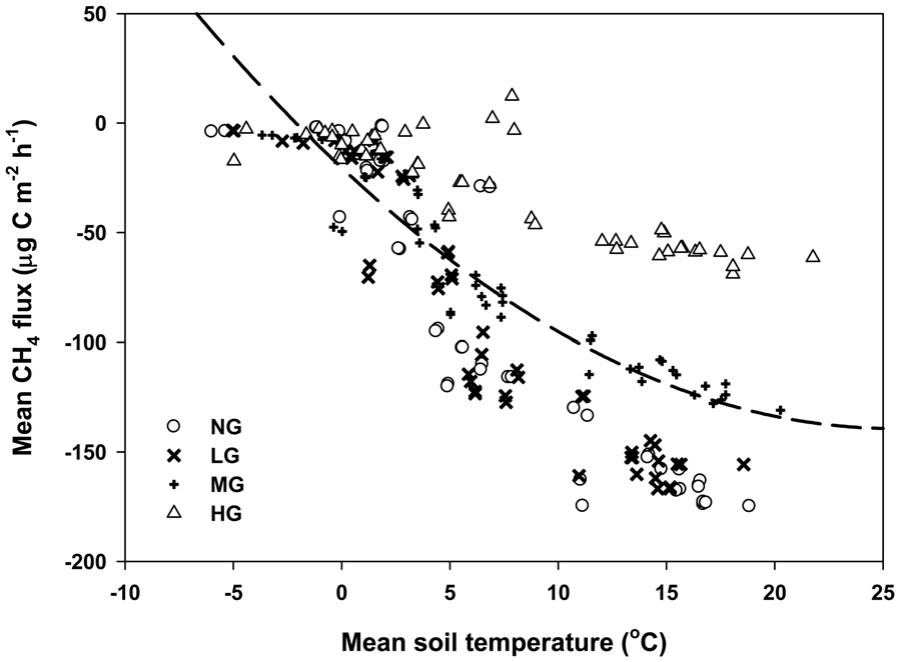
Dependency of CH_4_ fluxes on soil temperature (5 cm depth) under different stocking rates. NG, LG, MG, and HG are non-grazing, light grazing, moderate grazing, and heavy grazing, respectively.

**Figure 4:**
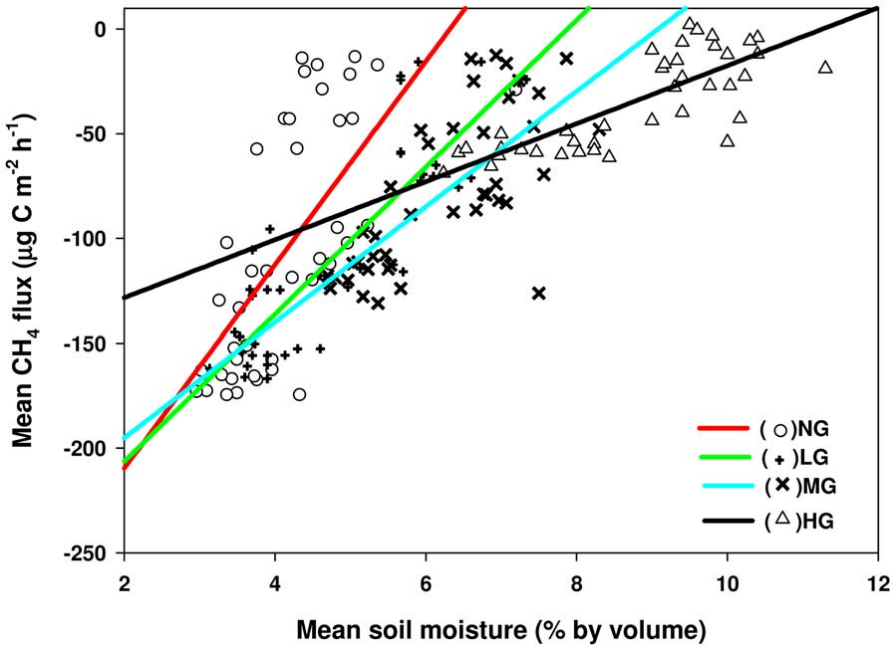
Dependence of CH_4_ fluxes on soil moisture (0–5 cm depth) under different stocking rates. NG, LG, MG, and HG are non-grazing, light grazing, moderate grazing, and heavy grazing, respectively. Each data point of daily CH_4_ flux measurement is the mean of three replicates.

The measured daily mean soil temperature (5 cm) and soil moisture (0–5 cm, for period II only) are illustrated in Figure 1 (A, B, E, F). The relationship between CH_4_ fluxes and soil temperature is best represented by a quadratic function [F(x) = 0.18x^2^−9.31x−20.39, R^2^ = 0.60, P<0.0001] (Figure 3 and Table 4). Soil moisture increased with an increase in stocking rate during the spring thawing period (Figure 1E, F). Using the linear regression function, we found that the soil moisture dependencies were different between the grazing areas (Figure 4 and Table 4). CH_4_ uptakes were negatively correlated with soil moisture in this study. The slope value (b) of the functions also indicated the difference of CH_4_ uptakes at the different grazing plots (Table 4).

**Table 4:** Parameters of the equations between daily CH_4_ fluxes (µg C m^−2^ h^−1^) and daily mean soil (5 cm) temperature (°C) or daily soil (0–5 cm) moisture (% by volume) at each site.

Items	a	b	c	R^2^	P
T [fitting with F = ax^2^+bx+c]					
	0.18	−9.31	−20.39	0.60	<0.0001
M [fitting with F = bx+c]					
NG	-	48.55	−306.77	0.47	<0.0001
LG	-	35.11	−276.59	0.75	<0.0001
MG	-	27.56	−250.23	0.51	<0.0001
HG	-	13.68	−156.11	0.59	<0.0001

T and M represent the soil temperature and soil moisture, respectively; NG, non-grazing; LG, light grazing; MG, moderate grazing; HG, heavy grazing.

## Discussion

### Impacts of freeze-thaw events on CH_4_ exchange

The most important natural sink of CH_4_ is in natural upland and forest soils, where methanotrophic activity occurs. The exchange of soil-atmosphere CH_4_ is comprised of two parts: CH_4_ produced by soil methanogenesis and atmospheric CH_4_ oxidation by soil [12]. Physiological activity of microorganisms is limited by temperature, moisture, physical structure, and nutrient pools of soil during the freeze-thaw period [9], [13], [14]. In our study, CH_4_ uptake was significantly influenced by freeze-thaw cycles in the desert grassland. This is inconsistent with a previous study conducted in a typical grassland area of Inner Mongolia [7]. Their results found that soil temperature was not a main constraint on CH_4_ uptake during the freeze-thaw period. We assume that the activity of microorganisms increased with the increase of environmental temperature. This is supported by Sharma et al. (2006) whose research in Southern Germany found that freeze-thaw causes significant physical and biological changes in the soil [13]. On the other hand, CH_4_ fluxes are also affected by different textured model soils. Low clay content in desert grassland soil is prone to CH_4_ uptake since it promotes methanotrophic activity of soils [15]. Further studies are required to elucidate the effect of grassland type on CH_4_ consuming microbes in soils during the freeze-thaw cycle.

### Stocking rate effects on CH_4_ exchange

The investigated areas acted as a sink for atmospheric CH_4_ during the freeze-thaw period in this study. This is in agreement with other studies conducted in other ecosystems [4], [7], [8], [16]. In our study, mean CH_4_ uptake during the measured period was 84.76 µg CH_4_-C m^−2^ h^−1^ (NG), which is significantly higher than in another study in an Inner Mongolian typical steppe [7]. Wang et al. (2005) reported CH_4_ uptake of 28±42 µg CH_4_-C m^−2^ h^−1^ and 22±19 µg CH_4_-C m^−2^ h^−1^ for non-grazed and grazed *Leymus chinensis* steppe during the non-growing season [17]. However, differences in grassland type, soil property, grazing duration, grazing density, measurement frequencies and method make it difficult to compare those results with ours.

Previous studies indicated that an increase in stocking rate induces a reduction in CH_4_ uptake [7], [8]. Simulating the effects of grazing management with the PaSim model, Soussana et al. (2004) also suggested that a decline in the GHG sink activity of managed steppes occurs with increased stocking density [18]. We also found a significantly negative correlation between stocking rate and soil CH_4_ uptake. However, our results indicate that LG induced a slight increase in CH_4_ uptake by soils compared to the NG areas during the spring thaw period. We assume that LG may enhance CH_4_ uptake by increasing the population size of CH_4_ oxidizing bacteria during the freeze-thaw period in the desert steppe. This is supported by research from a typical steppe in Inner Mongolia [19]. Similarly, Chen et al. (2011) also reported light-to-moderate (stocking rate ≤1 sheep ha^−1^ yr^−1^) grazing did not significantly change CH_4_ uptake compared with non-grazed typical steppes [20]. Our results are therefore likely to be pertinent in the context of the current debate on the global magnitude of CH_4_ emissions from agricultural ecosystems.

Two mechanisms are noteworthy with regard to the significant effect of HG on CH_4_ fluxes. 1) soil bulk density (0–10 cm) increased along the grazing gradient in our study (Table 1), which may result in a reduction of CH_4_ diffusion in soil due to reduced pore continuity under HG. This explanation was supported by Chen et al. (2011) who showed a significant linear dependence of topsoil air permeability (AP, cm s^−1^) on stocking rates (SR, sheep ha^−1^ yr^−1^; i.e., AP = −1.46SR+4.45) [20]; 2) although the nutrient pools (e.g. organic carbon, C to N ratio) of soils were hardly affected by HG in this study (Table 1), extraction from grazing animal had the higher potential for CH_4_ emission [21], which could offset CH_4_ uptake of soil in intensively grazed steppe.

Very low CH_4_ emissions were observed during the freeze-thaw period (Period I) in our study. This could be related to snowfall occurring before this experiment. The thaw of snow patches increased topsoil moisture content. A high moisture content of soil allows the development of methanogenic activity [15], [22]. This could be an important issue to address in efforts to improve estimates of the CH_4_ sink and source potential of desert steppe soils.

### Relationships between CH_4_ flux and other factors

Soil temperature and moisture content were considered as potential influencing factors on CH_4_ fluxes. Although some studies have reported that there is a strong relationship between soil temperature and CH_4_ uptake [4], [17], [20], in agreement with our findings, most studies found this relationship is limited by soil texture, bulk density, and soil moisture, placing greater importance with controls on gas diffusivity as the determining variable [7], [23].

Soil moisture increased along a grazing gradient during the freeze-thaw period (Period II) (Figure 1E, F), which could be caused by an increase in soil compaction due to the high stocking rate [24], leading to a decrease in soil moisture evaporation during the freeze thaw period. This indicates that the effect of grazing events on soil moisture is different between the growing season and the thawing period. An important factor determining CH_4_ uptake is gas transport resistance, which is influenced by soil wetness [7], [8], [17], [22]. This view is supported by our findings (Period II) that CH_4_ uptake decreased with an increase in soil moisture (Figure 4). Therefore, soil moisture is a major predicting variable for estimation of soil CH_4_ dynamics in desert grassland ecosystems.

The possible effects of factors other than soil temperature and moisture on CH_4_ uptake were not considered in this study. Consequently, we did not make observation for these other processes in our research area. However, they may produce great influences on CH_4_ uptake, and should not be excluded elsewhere. The practice of grazing sheep is a complex event in Inner Mongolian desert grasslands. A range of specific factors, such as fecal and urine deposition, shifts in plant rhizosphere exudation, shifts in plant species, and changes in soil structure and aerobicity can change soil characteristics [25]. Pol-van Dasselaar et al. (1998) reported that CH_4_ uptake may be promoted by reduced inorganic nitrogen content [22]. Aronson et al. (2010) also reported that smaller amounts of N tended to stimulate CH_4_ uptake while larger amounts tend to inhibit uptake by soil [26]. Therefore, further studies involving the effects of nitrogen, as well as long-term and intensive measurements of soil-atmosphere CH_4_ exchange are needed in desert grasslands.

### Conclusion

Our findings offer substantial evidence for differences in soil-atmospheric CH_4_ exchange during spring freeze-thaw cycles under different stocking rates in a desert steppe ecosystem. These results are in accordance with other findings that suggest the degree of steppe degradation should be considered for annual CH_4_ budgets. CH_4_ uptake by soil significantly decreases with an increase in stocking rates. LG can exert a considerable positive impact on CH_4_ uptake in the desert steppe at a regional scale. Hence, optimal grazing management practices could play a key role in controlling CH_4_ uptake in temperate desert steppes.
